# Supplementary material for: Synthesis of CNT@CoS/NiCo Layered Double Hydroxides with Hollow Nanocages to Enhance Supercapacitors Performance
Source: Nanomaterials (Basel). 2022 Oct 7;12(19):3509. doi: 10.3390/nano12193509 (PMC9565481; doi:10.3390/nano12193509)
Supplement: Supplementary file 1 [file nanomaterials-12-03509-s001.zip › nanomaterials-1930626-supplementary.pdf]

nanomaterials

Supplementary Materials

Synthesis of CNT@CoS/NiCo Layered Double Hydroxides with Hollow Nanocages to Enhance Supercapacitors Performance

Xiaoming Yue [1,*], Zihua Chen [1], Cuicui Xiao [1], Guohao Song [1], Shuangquan Zhang [1] and Hu He [2,*]

[1] Key Laboratory of Coal Processing and Efficient Utilization (Ministry of Education) and School of Chemical Engineering and Technology, China University of Mining and Technology, Xuzhou 221116, China
[2] School of Resources and Geosciences, China University of Mining and Technology, Xuzhou 221116, China
* Correspondence: yuexiaoming_cumt@126.com (X.Y.), hehu@cumt.edu.cn (H.H.)

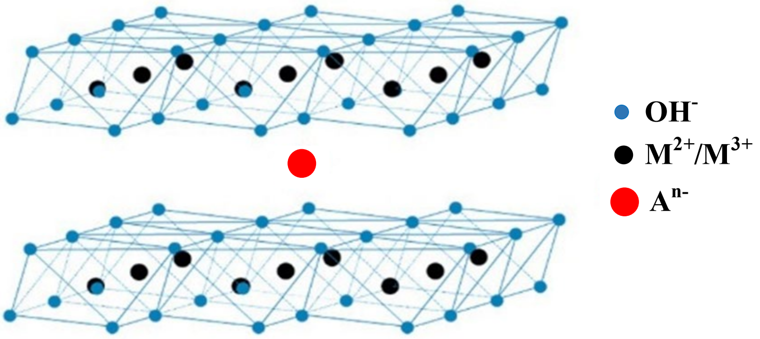

Figure S1. The structure model of NiCo-LDH.

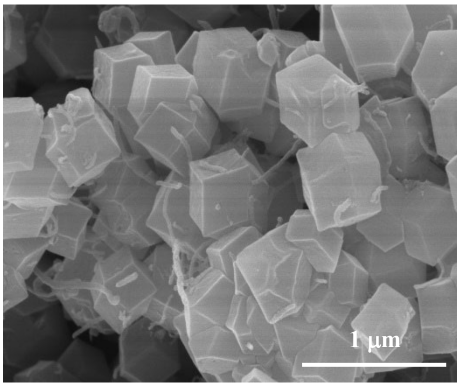

Figure S2. SEM image of CNT/ZIF-67.

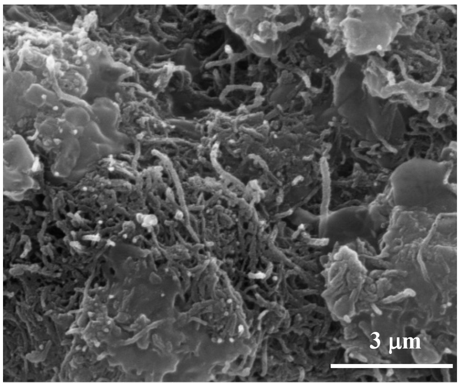

Figure S3. SEM image of CNT/NiCo-LDH.

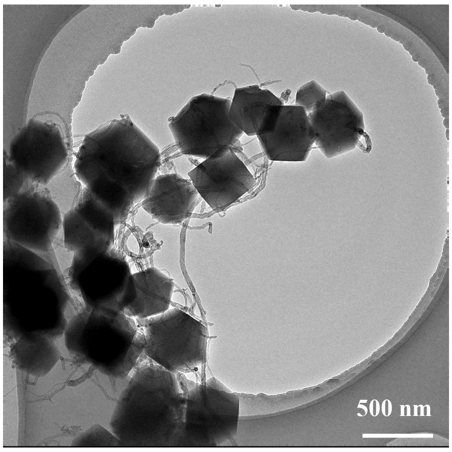

Figure S4. TEM image of CNT/ZIF-67.

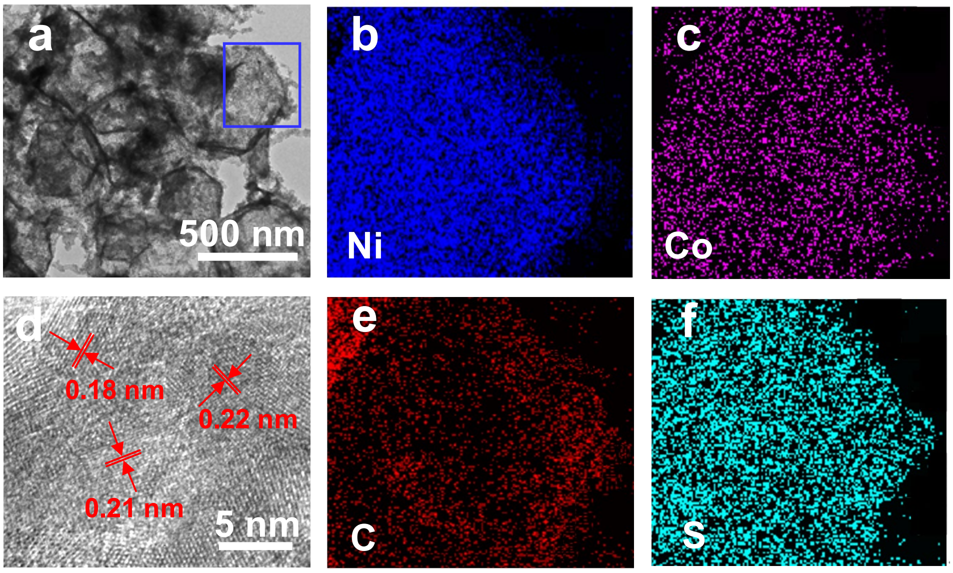

Figure S5. (**a**) TEM image of C@CS/NCL-100, (**b**, **c**, **e**, **f**) TEM-EDS element mapping images of C@CS/NCL-100, (**d**) HRTEM image of C@CS/NCL-100.

Table S1. A list of NiCo-LDH materials in the application of supercapacitors, compared to that obtained in this work.

Materials (three-electrode system)	Electrolyte	Specific capacitance (Potential range vs. Hg/HgO)	Electrodes (asymmetric supercapacitors)	Energy density (Wh kg^{-1})/Power density (W kg^{-1})	Ref.
$Ni_{0.28}Co_{0.72}(OH)_2$	6 M KOH	1255.0 F g^{-1} (0.05-0.55 V,1 A g^{-1})	$Ni_{0.28}Co_{0.72}(OH)_2$//AC	20.6/3930	[1]
NiCo-LDH (ZIF-67 derived)	2 M KOH	2369.0 F g^{-1} (0-0.5 V,0.5 A g^{-1})	NiCo-LDH//AC	21.28/3741.0	[2]
PNT@NiCo-LDH (ZIF-67 derived)	6 M KOH	1448.2 F g^{-1} (0-0.5 V,1 A g^{-1})	PNT@NiCo-LDH//AC	64.4/800	[3]
CoSx/NiCo-LDH (ZIF-67 derived)	2 M KOH	1562 F g^{-1} (0-0.45 V,1 A g^{-1})	CoSx/NiCo-LDH//AC	35.8/800	[4]
CNT@ NiCo-LDH (MOF derived)	6 M KOH	1159.2 F g^{-1} (0-0.5 V,1 A g^{-1})	CNT@NiCo-LDH//CNT@NC	37.4/750	[5]
CNT@CoS/NiCo-LDH	6 M KOH	2794.6 F g^{-1} (0-0.5 V,1 A g^{-1})	CNT@CoS/NiCo-LDH//AC	31.38/3750	This work

References:

1. Tang, Y.; Liu, Y.; Yu, S.; Guo, W.; Mu, S.; Wang, H.; Zhao, Y.; Hou, L.; Fan, Y.; Gao, F. Template-free hydrothermal synthesis of nickel cobalt hydroxide nanoflowers with high performance for asymmetric supercapacitor. *Electrochim. Acta* **2015**, 161, 279-289.
2. Wang, M.; Feng, Y.; Zhang, Y.; Li, S.; Wu, M.; Xue, L.; Zhao, J.; Zhang, W.; Ge, M.; Lai, Y.; Mi, J. Ion regulation of hollow nickel cobalt layered double hydroxide nanocages derived from ZIF-67 for High-Performance supercapacitors. *Appl. Surf. Sci.* **2022**, 596, 153582.
3. Zang, Y.; Luo, H.; Zhang, H.; Xue, H. Polypyrrole Nanotube-Interconnected NiCo-LDH Nanocages Derived by ZIF-67 for Supercapacitors. *ACS Appl. Energ. Mater*. **2021**, 4(2), 1189-1198.
4. Guan, X.; Huang, M.; Yang, L.; Wang, G.; Guan, X. Facial design and synthesis of CoSx/Ni-Co LDH nanocages with rhombic dodecahedral structure for high-performance asymmetric supercapacitors. *Chem. Eng. J.* **2019**, 372, 151-162.
5. Niu, H.; Zhang, Y.; Liu, Y.; Xin, N.; Shi, W. NiCo-layered double-hydroxide and carbon nanosheets microarray derived from MOFs for high performance hybrid supercapacitors. *J. Colloid Interf. Sci.* **2019**, 539, 545-552.
